# Enhancing the Low-Frequency Induction Heating Effect of Magnetic Composites for Medical Applications

**DOI:** 10.3390/polym12020386

**Published:** 2020-02-08

**Authors:** Ziyin Xiang, Khao-Iam Jakkpat, Benjamin Ducharne, Jean-Fabien Capsal, Jean-François Mogniotte, Patrick Lermusiaux, Pierre-Jean Cottinet, Nellie Della Schiava, Minh Quyen Le

**Affiliations:** 1 INSA-Lyon, Electrical Department, Univ. Lyon, LGEF, Ladoua Campus, EA682, F-69621 Villeurbanne, France; ziyin.xiang@insa-lyon.fr (Z.X.); jamejakkpat@gmail.com (K.-I.J.); benjamin.ducharne@insa-lyon.fr (B.D.); jean-fabien.capsal@insa-lyon.fr (J.-F.C.); jean-francois.mogniotte@insa-lyon.fr (J.-F.M.); pierre-jean.cottinet@insa-lyon.fr (P.-J.C.); nellie.della-schiava@chu-lyon.fr (N.D.S.); 2 HYBRIA Institute of Business and Technologies, Écully Campus, 69130 Écully, France; 3 Université Claude Bernard Lyon 1 (Univ. Lyon), 8 Avenue Rockefeller Lyon, F-69621 Villeurbanne, France; patrick.lermusiaux@chu-lyon.fr; 4 Groupement Hospitalier Edouard Herriot, 69003 Lyon, France

**Keywords:** ferromagnetic composites, magnetic particles, hysteresis loss, low-frequency induction heating, thermal stability, thermal transfer modeling, treatment in varicose veins, medical applications

## Abstract

This study aims to enhance the low-frequency induction heating (LFIH) effect in a thermoplastic polymer doped with iron oxide magnetic particles, which are promising candidates for several medical applications thanks to their confirmed biocompatibility. Two main approaches were proposed to successfully boost the heating ability; i.e., improving the magnetic concentration of the composite with higher filler content of 30 wt %, and doubling the frequency excitation after optimization of the inductor design. To test the magnetic properties of the ferromagnetic composite, a measurement of permeability as a function of temperature, frequency, and particle content was carried out. Thermal transfer based COMSOL simulations together with experimental tests have been performed, demonstrating feasibility of the proposed approach to significantly enhance the target temperature in a magnetic composite. These results are encouraging and confirmed that IH can be exploited in medical applications, especially for the treatment of varicose veins where local heating remains a true challenge.

## 1. Introduction

Induction heating (IH) is a noninvasive heating technology based on inducing an alternating (AC) magnetic field in a medium to be heated [1]. When an object is placed in this field, two heating effects occur: hysteresis losses and eddy-current losses. The first effect only appears in ferromagnetic materials such as iron, nickel, and cobalt, due to the friction between the particles when the material is being continuously magnetized in different directions. This phenomenon is associated with the wall domain movement that predominates in high-frequency excitation or ferromagnetic nano/micro particle heating. A higher magnetic oscillation frequency results in faster particle movement, which causes more friction, and thus more heat. The second effect is Joule heating in any conductive material because of the electric currents induced by the fluctuating magnetic field. Both effects result in heating of the treated object, but the second is the main heat source in IH processes.

Compared to other classical heating techniques, such as flame heating, resistance heating, and traditional ovens or furnaces, IH offers fast, clean, and precise temperature control in a contactless and efficient way. It is one of the most preferred heating technologies in industrial [2], domestic [3], and medical applications [4]. Although the process parameters in many industrial and domestic applications are already well-known, there are still some issues that need further optimization: heating of low-resistivity materials, accurate heating of biological tissues, faster design for complex IH load geometries, and accurate 3D FEA simulation of the whole IH system [5]. The third major area of IH is medicine, and this field is not as mature as industrial or domestic applications. It has lately attracted a great deal of research interest. IH was initially applied only in manufacturing and sterilization of many surgical instruments because it is a clean, fast, and portable heat source.

IH has recently started to be introduced in minimally-invasive hyperthermia as a cancer treatment therapy by inducing a temperatures of about 41–45 °C to the cancerous cells [6,7]. In order to precisely deliver the power to the tumor, a ferromagnetic material is usually placed in the area to be treated. This technique efficiently destroys cancer tissue while minimizing the damage to the surrounding healthy cells. Moreover, this local treatment can markedly reduce pain compared to chemotherapy. The frequencies used for hyperthermia are usually inside the margins of radiofrequency (i.e., hundreds of kHz to few MHz) [8,9] or microwaves (hundreds of MHz to 10 GHz); i.e., non-ionizing radiation frequencies. However, frequencies over 100 kHz can produce significant absorption of energy in the body when the procedure is longer than it should be. As a result, exposure time to high frequency magnetic field is an important factor that must be considered to avoid side effects.

We recently [10] reported a new technique of inductively-heated ferromagnetic composite-based acrylonitrile butadiene styrene (ABS) thermoplastic filled with iron oxide (Fe_3_O_4_) microparticles. There was a significant increase in the temperature of this under low frequency (LF) magnetic excitation of only few kHz. This frequency was drastically smaller than the one used in most IH applications—especially hyperthermia therapy. The use of LF magnetic sources is safer, simpler, cheaper, and more space efficient. These results [10] were very promising and showed the value of LF inductive material for minimally invasive endovascular treatment of varicose veins.

The magnetic particles used in this prior work are non-toxic, injectable, and accumulate in the target tissue or organs [11]. The concept is to insert a biocompatible composite into an abnormal vein and cauterize (burn) the tissue under a high temperature via an external AC magnetic field. Due to their ferromagnetism properties, only particles under magnetic excitation would be active, leading to local heating of the diseased vein without damaging the surrounding healthy tissue. The magnetic source was implemented outside the patient with no physical connection to the composite introduced into the vessel. Here, low-frequency induction heating (LFIH) leads to easier procedure with respect to currently existing techniques based on endovenous thermal ablation (ETA)-like endovenous laser ablation (EVLA), endovenous steam ablation (EVSA), and radiofrequency ablation (RFA). Moreover, this technique uses ferromagnetic composites involving ABS polymer matrix doped with magnetic filler, so it can be combined with additive manufacturing, also known as 3D printing [12,13]. This approach allows the fabrication of smart materials with various special shapes and sizes, which will be suitable for multiple sorts of veins and can overcome the drawbacks of current ETA therapies. Additionally, a linear dependence of magnetic strength on the rotational motor frequency provides an easy way for LFIH to vary energy delivery during a medical procedure. This is impossible in the case of RFA [14]. Finally, LFIH is a good alternative treatment to produce minimal undesired effects on patients.

Here, we provide additional analysis based on the LFIH effect as well as significant improvements in terms of heating performance with respect to the device reported previously [10]. A target composite made of 17% vol. of Fe_3_O_4_ particles used in previous work was heated up to 65 °C via an AC magnetic excitation of 2300 Hz, which was far from the prerequisite imposed in venous insufficiency procedures where a goal temperature between 100 to 120 °C must be achieved. Therefore, the main objective of this study was to enhance the LFIH mechanism so that it can match the medical requirements more closely. Several solutions can overcome the technological problems described previously [10], such as increasing the magnetic concentration of the composite up to 30% and increasing the frequency excitation by optimization of inductor design. Satisfactory results have been achieved with an important heating temperature close to 100 °C. These results demonstrate the reliability of the proposed approach. Furthermore, this work revealed that IH efficiency not only depends on the magnetic content and frequency excitation but also on the nature of magnetic particle as well as size and shape of the whole composite. Additionally, this study demonstrates the stability of the magnetic characterization of the fabricated material via inductance measurements from −10 °C to 100 °C. COMSOL simulations were also performed and show strong convergence between experiment and modeling. A further issue for future research will be more accurate control of the heating process, including target temperatures and specific localization. This is a vital requirement for IH systems, especially for clinical environments.

## 2. Material Fabrication

The magnetically-reinforced material had to be biocompatible, because the final goal of this work was medical application. Iron oxide (Fe_3_O_4_) particles have been extensively studied in the last few decades for several biomedical applications, such as magnetic resonance imaging, magnetic drug delivery, and hyperthermia [15]. Additionally, this material has been widely studied in recent years due to its interesting magnetic properties, making it potentially interesting for numerous applications [16]. Regarding the polymer matrix, the thermoplastic ABS (acrylonitrile butadiene styrene) was considered a suitable choice due to its widespread commercial use—particularly in injection molding and 3D printing [17].

Fe_3_O_4_ spherical powders 5 µm in of diameter were purchased from SIGMA-ALDRICH. This particle size was chosen to achieve the best trade-off between the material dispersion and mechanical properties of composite. Actually, too small a size for particles, such as a nano-scale size, can lead to critical dispersion when elaborating a polymer solution with magnetic powder, particularly in the case of high filler content (e.g., greater than 20% volume). Therefore, nanoparticles are generally used in the fabrication of low particle content. On the other hand, too big of a particle size can make a composite more rigid and fragile, leading to a significant change in mechanical behavior of the whole sample. Furthermore, according to the point of view of magnetic characteristics, using of microparticles instead of nano ones allows one to create multi-domain wall movement, improving hysteresis loss, and thus heating energy. Indeed, a single domain nanoparticle can induce heat by another loss mechanisms, called Néel and Brownian relaxations by which the magnetization of magnetic nanoparticles can relax back to their equilibrium position after the application of magnetic field is removed. As a result, single domain NPs have large specific absorption rates and possibly produce heat under low magnetic field amplitude, but very high frequency is needed to favor relaxation losses. Contrarily, multi-domain particles require a larger field amplitude for extensive heating, but can be heated under much lower dynamics. In this study, we focus only on the low frequency IH effect of around few kHz. Therefore, the use of magnetic particles at the order of few µm is more adequate to facilitate hysteresis losses caused by multi-domain wall movement.

**Figure 1:**
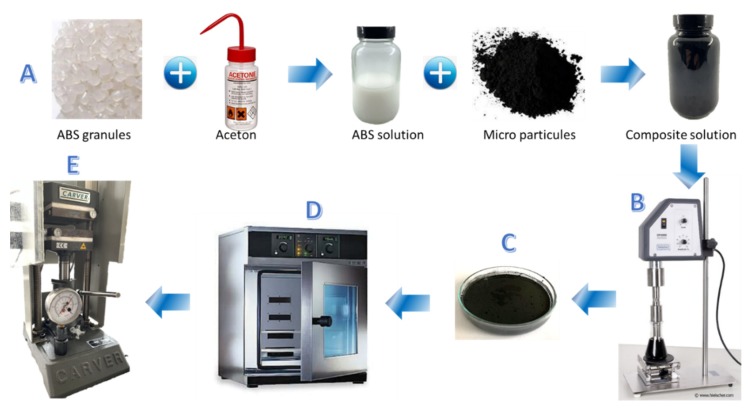
The fabrication process of the ferromagnetic composite in ABS consists of 5 main steps: (**A**) preparation of the composite, including iron powder incorporated in ABS solution; (**B**) ultra-sonication for achieving a homogenous solution; (**C**) deposition of the solution into an evaporating dish; (**D**) sample was heated in an annealing oven to efficiently evaporate the solvent; and (**E**) the composite was hot pressed under high pressure and temperature to ensure a perfectly compact homogenous block with a desired shape by using a specific mold.

The elaboration procedure of the ferromagnetic composite is illustrated in Figure 1. First, ABS granules were dissolved in acetone with vigorous stirring at room temperature for 2 h until the ABS was completely liquefied. Second, Fe_3_O_4_ powders were added and stirring was continued for 1h to achieve a perfectly homogeneous solution (Figure 1A). The volumetric content of Fe_3_O_4_ in the ABS varied from 3% to 30%, which was higher than our prior work [10,18] where the particle composition was limited to 17%. Other studies showed that increasing the magnetic concentration was an easy way to enhance the induction heating performance [19]. In this work, we improved the fabrication process to achieve composites with superior magnetic properties. To some extent, the particle percentage is limited at 30% to avoid heterogeneity and percolation thresholds that can occur at a high filler content. Considering that the particle distribution was homogeneous, each particle was assumed to be electrically insulated. Such electrical insulation will prevent the formation of macroscopic eddy currents. Consequently, the ferromagnetic losses will be limited to the domain wall motions resulting in microscopic eddy currents and local induction heating effects.

The iron powder was dissolved in the ABS matrix via ultrasound (Hielscher Ultrasonic Processor UP400S, Teltow, Germany) (Figure 1B). Subsequently, the solution was precipitated in ethanol within 30 min to freeze the composite in a good dispersion state and avoid sedimentation of the particles in the polymer solution. Next, the obtained solution was transferred into an evaporating dish, and the collected supernatant liquid was withdrawn (Figure 1C). The sample was then put in the oven (Memmert Typ: V0 400, Schwabach, Germany) at 56 °C (corresponding to the acetone volatilization temperature) for 2 h to totally evaporate the solvent (Figure 1D). The powdered composite was then slowly hot pressed (Figure 1E) at 220 °C under a pressure of 1300 PSI in a hydraulic press (CARVER 3851CE, Wabash, IN, USA). This temperature is close to the melting temperature of ABS (≈210 °C–230 °C) to ensure a perfectly compact homogenous block.

**Figure 2:**
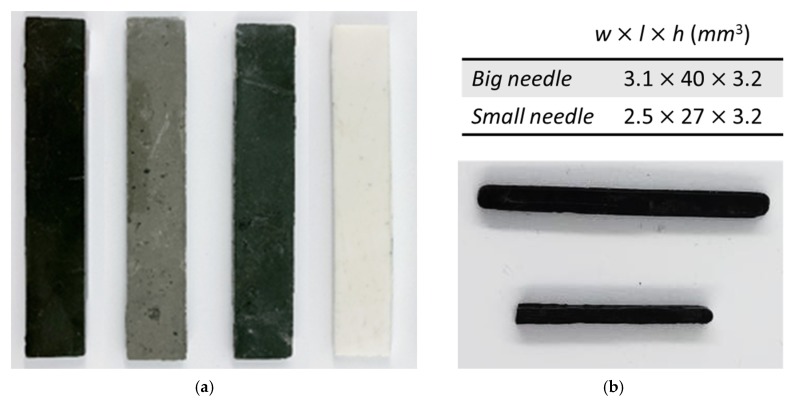
(**a**) Rectangular samples (from left to right): Three ferromagnetic composites filled with iron oxide, nickel, or manganese; pure ABS is also included. (**b**) Samples of needle shape.

Experimental tests are described in Section 3. For these tests, samples were made in a rectangular shape with dimensions of 60 × 14 × 4 mm. In order to better justify the choice of a pertinent ferromagnetic component as iron oxide Fe_3_O_4_, other common elements of low cost and good magnetic properties, such as nickel (Ni) and manganese zinc (Mn-Zn), were used in our fabrication process. Figure 2a illustrates four different materials, including the ferromagnetic composite reinforced by Fe_3_O_4_, Ni, or Mn-Zn particles, and pure ABS is a control. The composite depicted on Figure 2a is too big for the endovenous procedure. Thus, a second series of samples was designed in a needle shape comprised of two different sizes; i.e., the big one with dimensions of 3.1 × 40 × 3.2 mm^3^, and the small one with 2.5 × 27 × 3.2 mm^3^ (Figure 2b). Both needle samples nicely match typical vein diameters of 4–5 mm.

## 3. Results and Discussions

### 3.1. Experimental Setup

**Figure 3:**
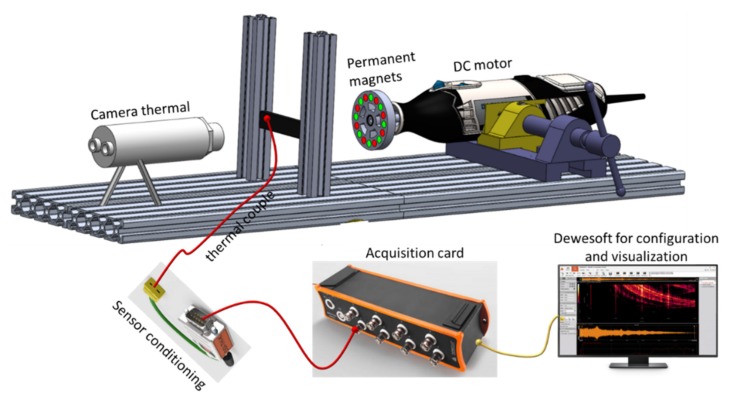
Experimental setup of the induction heating (IH) equipment.

**Figure 4:**
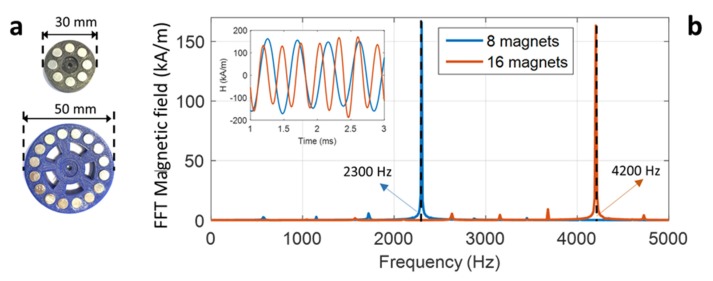
(**a**) Eight-magnet and 16-magnet inductors. (**b**) FFT spectra of magnetic field driven by the two types of inductors under 35 kRPM motor speed. Inset: Time evolution of the magnetic excitation induced from measurement with the H-coil.

A specific experimental test-bench was developed to validate the IH effect (Figure 3). A thermocouple was coated on the sample via an adhesive to measure the temperature of heat area corresponding to the magnet’s passage. To obtain a more accurate temperature image, a thermal camera (Optris Xi400, Berlin, Germany) was used during the experiment. All data were recorded in real time through a Krypton card (DEWESoft, Ivry-sur-Seine, France). To generate a significant AC magnetic field excitation, a magnetic inductor was assembled to a DC drill motor at variable speed. Two kinds of magnetic inductors were employed: One consisted of eight cylindrical permanent magnets already mentioned in [10,18], and the other had a double of identical permanent magnets (i.e., 16) as developed in this study (cf. Figure 4a). The goal of the latter was to achieve higher magnetic frequency excitation. The pole distribution of the permanent magnets was alternatively southern and northern, enabling the production of a sinusoidal magnetic excitation whose frequency was fourfold increased (with eight magnets) or eightfold increased (with 16 magnets) with respect to the one driven by the DC motor.

The current test bench has a maximum rotating speed of 35 kRPM, and the highest theoretical frequencies generated by the two inductors are 2300 Hz and 4600 Hz, respectively. In reality, the 8-magnet source can reach an AC magnetic field of 2300Hz, as expected, but the one driven by the 16-magnet source leads to a frequency of 4200 Hz. This was slightly lower than the estimated value (see the spectra in Figure 4b). The fact is that the new inductor containing a double of permanent magnets becomes heavier, leading to the increase rotational inertia. As a result, more torque was exerted on the motor, somehow reducing the speed.

### 3.2. Experimental Result

**Figure 5:**
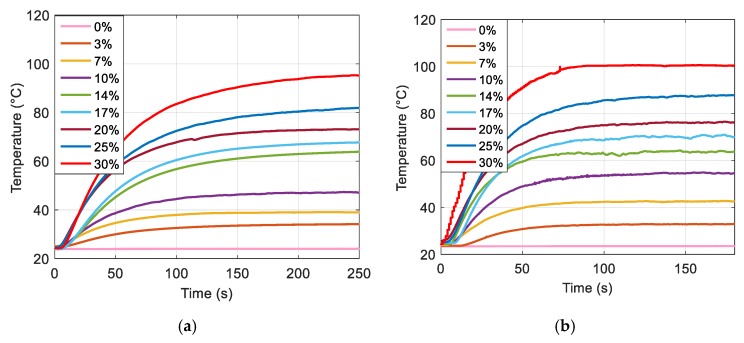
Time evolution of the temperature at different particle fraction using (**a**) an 8-magnet inductor and (**b**) a 16-magnet inductor.

Figure 5a,b displays the temperature vs. time variations of ferromagnetic composites with different volume concentrations from 0% to 30% powered under magnetic sources with two different frequency excitations. In both cases, the temperature remained constant for the pure ABS thermoplastic, but it increases for samples with higher magnetic powder content. Indeed, a polymer filled with sufficient ferromagnetic particles led to substantially improved hysteresis losses, giving rise to a drastically increased magnetic power density, thereby boosting the induction heating effect.

**Figure 6:**
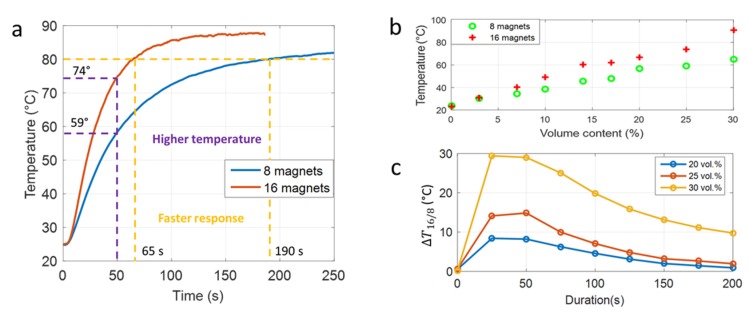
(**a**) Temperature evolution of the 25% Fe_3_O_4_ composite using two different inductor excitations: (**b**) Temperature of all iron oxide composites with different concentrations after 50 s. (**c**) Temperature variation of two magnetic sources ($∆T_{16/8}$) as a function of duration.

For all samples, the 16-magnet inductor results in higher temperature variation as well as faster response. Figure 6a shows a composite doped with 25% Fe_3_O_4_, which was inductively heated at 59 °C and 74 °C after 50 s via the 8-magnet source and 16-magnet source, respectively. Furthermore, to reach the target temperature (e.g., 80 °C), the new device only needed 65 s, which is three-fold faster. Figure 6b illustrates the temperature behavior in terms of volume fraction of the composite under two different magnetic frequency excitations with recording after 50 s. The results show a linear relationship between the heating temperature and the magnetic particle content. Interestingly, the gap between the two curves increases as a function of the iron oxide ratio, showing the benefit of using high filler content ferromagnetic composite to improve hysteresis losses inside each particle. Figure 6c displayed the temperature variation ($∆T_{16/8}=T_{16\;magnets}-T_{8\;magnets}$) of three samples (i.e., 20% vol., 25% vol., 30% vol.) driven by the two magnetic sources as a function of the exposure time. The results confirmed the decreasing behavior of $∆T_{16/8}$ with longer heating durations (above 50 s). The value of $∆T_{16/8}$ becomes significant under short operation times (25–50 s). It then quickly drops after more than 100 s. Consequently, the newly designed inductor shows a further advantage over the former—especially in medical applications where a fast response time is mandatory.

Thermal considerations are important for IH component design because the materials can be heated by external sources or by their own energy losses. In order to check the magnetic performance, the fabricated composites were put into an oven (VOTSCH Industrietechnik VT 7004, Balingen, Germany) from −10 °C to 100 °C. After reaching a stable temperature, inductance measurements were performed using a high precision LCR meter (E4980A Keysight, California, US). All samples were manually wired with 150 turns in a single layer, which was sufficient to get a satisfactory inductance value. The operating frequency range of 1 kHz to 1 MHz was chosen to fit with most of the typical IH applications. Considering that the relative magnetic permeability of the pure ABS is uniform regardless the variation of temperature and/or frequency, the composite with the same dimensions can be deduced according to the following Equation:

(1) $$\mu_{r}=\frac{L_{composite}}{L_{ABS}}$$

where *L_composite_* and *L_ABS_* denote the inductance of the filler composite and the pure ABS, respectively.

**Figure 7:**
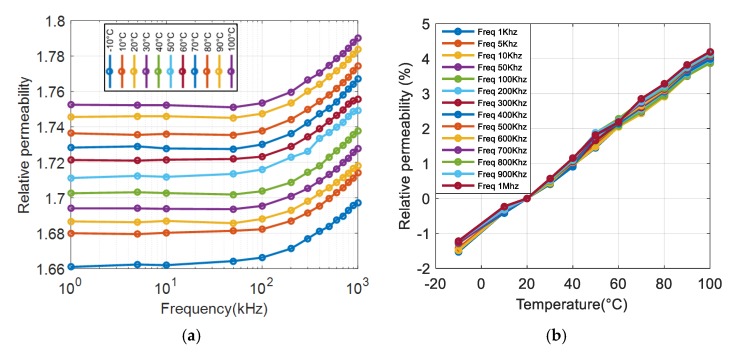
(**a**) Relative permeability spectra of the 30% Fe_3_O_4_ composite under different temperatures. (**b**) Relative permeability change (%) of the 30% Fe_3_O_4_ composite as a function of temperature.

Figure 7a shows that the magnetic permeability of the composite elaborated with 30% vol. iron oxide is almost constant until a frequency of 100 kHz, confirming the high potential of the developed materials for LFIH use. A small increase of the permeability was observed for all samples above 100 kHz to 1 MHz (i.e., around 1%–2%). This increase was not due to the intrinsic magnetic properties of the material but rather was caused by the self-resonant-frequency effect (SRF) of the wire-wound inductor. This phenomenon principally stems from parasitic capacitance in parallel to the inductor, which is a result of the individual turns of the coil being close to one another. The wired sample only acts like a pure inductor under a frequency lower than the SRF at which the impedance becomes very high, leading to imprecise inductance measurements of the LCR meter. Another method using more complex model (RLC instead of RL) was further investigated to accurately determine the permeability at very high frequency. This issue is considered to be out of the scope in this study because the operating frequencies are relatively low; i.e., less than 5 kHz.

Figure 7b displays the relative permeability change (i.e., Δ*μ_r_* (*T*)) as given in Equation (2) of the 30% Fe_3_O_4_ composite versus temperature.

(2) $$∆\mu_{r}(T)=\frac{∆\mu_{r}(T)-∆\mu_{r}(T_{amb})}{∆\mu_{r}(T_{amb})}$$

Here, Δ*μ_r_* (*T_amb_*) is the temperature of the composite at ambient temperature, which in our case equals 20 °C.

**Figure 8:**
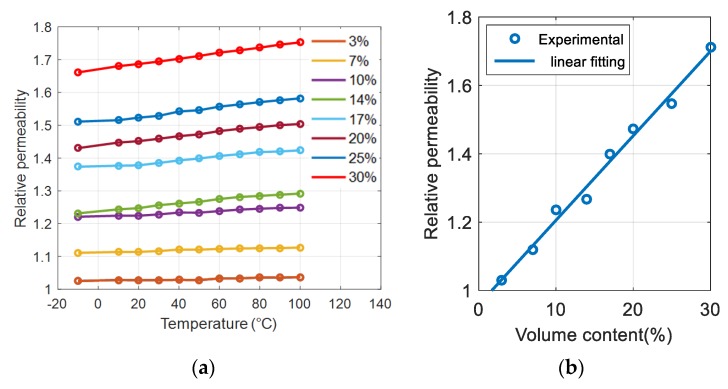
Relative permeability at 1 kHz as a function of (**a**) temperature and (**b**) volume content.

Figure 7b shows that under the entire frequency range, the relative permeability variation of the sample filled with 30% iron oxide is relatively small (no more than several percent), even at temperatures up to 100 °C. The typical changes in permeability over temperature for different filler contents were revealed in Figure 8a: All composites are stable at this temperature range. Interestingly, higher magnetic concentration in the composite led to greater increase in permeability with temperature; e.g., 5% for the 30% sample as opposed to 1% for the 3% sample. The result in Figure 8b highlights that the relative permeability linearly increases with the ferromagnetic particle contents, which is consistent with the literature [20].

**Figure 9:**
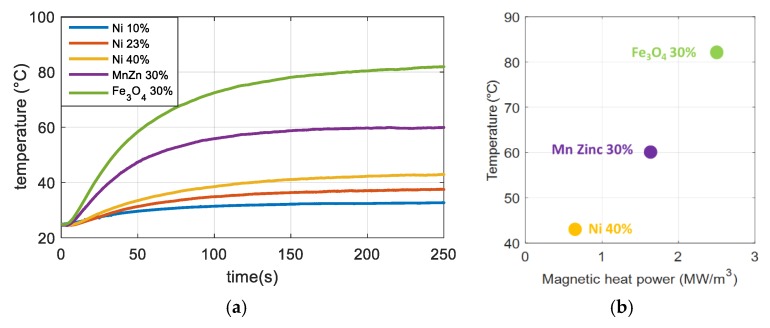
(**a**) Temperature versus time for composites filled with different types of ferromagnetic particles. (**b**) Temperature in terms of modeled magnetic heat power for Fe_3_O_4_, Ni, and Mn Zinc composites at a magnetic field of 160 kA/m amplitude and 2300 Hz frequency.

In order to better justify the choice of iron oxide, we compared the results with other magnetic particles, such as Ni and MnZn. These latter two materials are commonly used for inductor components due to their low cost, commercial availability, and high magnetic properties. Figure 9a displays the temperature evolution over 250 s for different composites of 30% Fe_3_O_4_; 30% Mn Zinc; and 10%, 23%, and 40% Ni. The results confirmed that the iron oxide material—thanks to its important hysteresis area—leads to the best temperature response with respect to the Ni and Mn Zinc. Indeed, the higher magnetic properties of the Fe_3_O_4_ allow it to efficiently drive magnetic flux inside the particles, thereby allowing for a significant increase in the induction heating effect. Figure 9b shows the density heat power under a magnetic field of 160 kA/m amplitude and 2300 Hz frequency estimated based on COMSOL. This result has a maximum value for the composite filled with 30%Fe_3_O_4_; i.e., corresponding to 2.4 MW/m^3^ as opposed to 1.7 MW/m^3^ for the 30% Mn Zinc and 0.7 MW/m^3^ for the 40% Ni. More details about the thermal transfer via COMSOL modeling have been published [10].

**Figure 10:**
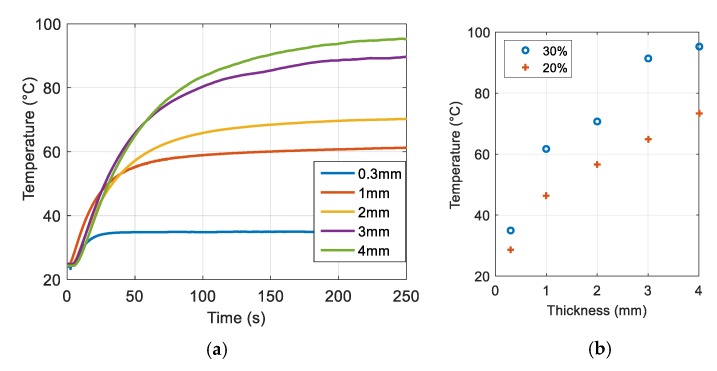
(**a**) Temperature versus time of 30% iron-oxide composites with different thicknesses. (**b**) Maximum temperature of the 20% and 30% samples as a function of thickness for the 8-magnet sources.

Figure 10a shows the temperature evolution versus time of the 30% iron-oxide composites with the same surfaces but different thicknesses from 0.3 mm to 4.0 mm. As expected, the temperature change is moderate for the thin composite film (0.3 mm), where only a 10 °C increase has been recorded. In contrast, samples with 3 mm or 4 mm thickness have a much thicker inductive temperature variation, showing that the IH effect strongly depends on the material’s volume (or thickness). Considering that the magnetic power density of a given composite is constant (see Figure 9b), an increase in volume leads to enhanced heating power. Thus, this increases the temperature change. This result was highlighted based on the increasing trend between the temperature and the sample’s thickness, as displayed on Figure 10b. However, a higher volume (or thickness) can result in an increase in the response time, which is one of the critical parameters that should be minimized to meet the medical requirements [10].

## 4. Ferromagnetic Composite Enhancement: Toward Medical Application

**Figure 11:**
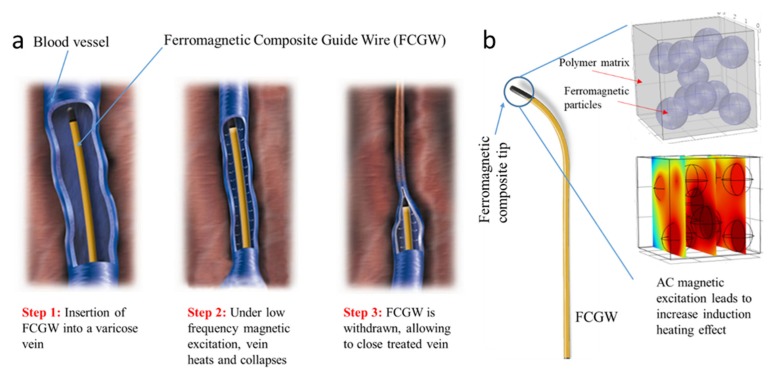
Working principal of ferromagnetic composite guide wire (FCGW) for varicose vein treatment. (**a**) Three principal steps in the procedure; (**b**) FCGW-design-based iron oxide composite.

Figure 11a illustrates the working principal of ferromagnetic composite guide wire (FCGW) for endovenous thermal ablation (ETA). This is an outpatient procedure and an alternative to surgical ligation and stripping for varicose veins. Here, magnetic excitation is applied through the patient’s epidermis to deliver heat and seal off targeted blood vessels. The development of such a concept for ETA therapy offers many advantages to patients compared to the traditional surgery, such as a shortened recovery period, less pain, and no scarring. The procedure consists of three principal steps, as described on Figure 11a. First, after using ultrasound to map the course of the treated vein, the surgeon inserts the FCGW through a small incision into the diseased vein, threading it through the blood vessel into the groin area. Second, a magnetic field is delivered to a target element heating and contracting the collagen within the walls of the vein until they shrink and disappear. The vein is thus treated in segments as the FCGW is gradually inched back down towards the incision. Finally, when the entire vein has been ablated, the blood flow is automatically rerouted through healthier adjacent veins, restoring healthy circulation and reducing swelling. The ablated vein becomes scar tissue and is absorbed by the body [21]. Figure 11b shows the guide wire design where the tip is made up of ferromagnetic-composite-based iron oxide particles embedded in a thermoplastic ABS matrix.

In order to integrate them with medical tools, the ferromagnetic devices were elaborated into a needle-like shape (cf. Figure 2b). Two kinds of samples with identical thicknesses (3.2 mm) but different surface exposures to magnetic sources were selected to better analyze the IH performance in terms of the material’s geometry. The selected dimensions of these two samples are adaptable to varicose vein diameters (4 and 5 mm).

**Figure 12:**
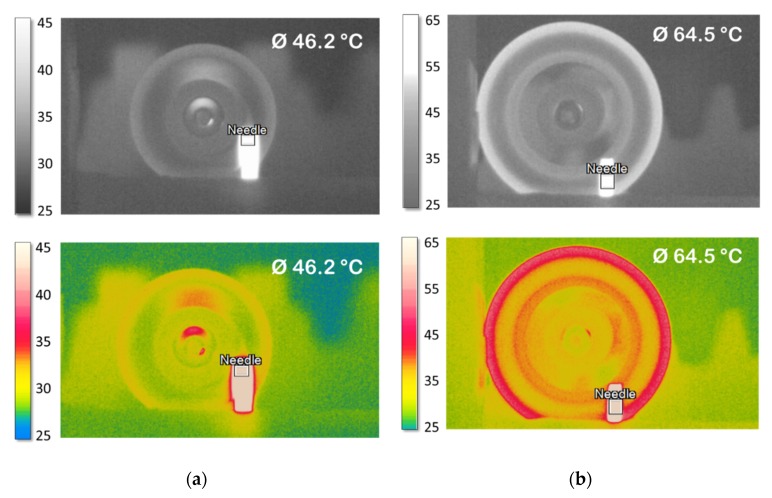
LFIH observation based thermal camera for big needle composite filled with 30% ferromagnetic particles using (**a**) the 8-magnet inductor, and (**b**) the 16-magnet inductor.

Figure 12a,b illustrates thermal camera imaging (in both colored and black and white resolutions) of the big needle doped with 30% ferromagnetic particles—these panels were powered by the 8- and the 16-magnet inductors, respectively. The target temperature of the composite was 46 °C under an AC magnetic field of 160 kA/m amplitude and 2300 Hz frequency via the 8-magnet source. The same magnetic strength with higher applied frequency (4200 Hz) was delivered from the 16-magnet. A significant increase in temperature was recorded of 65 °C.

**Figure 13:**
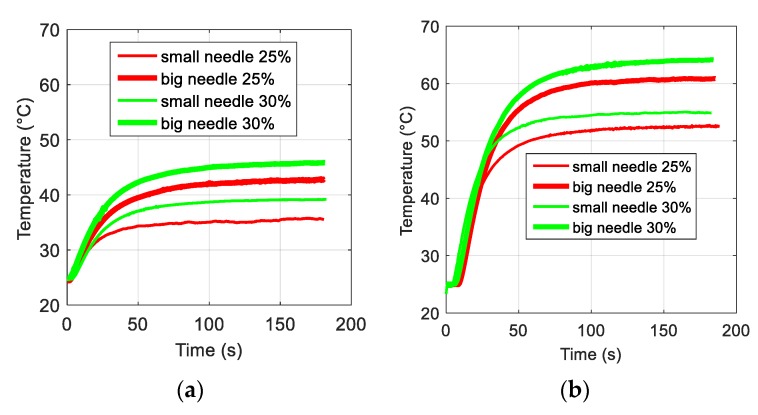
(**a**) Temperature versus time for needle composites filled with 25% and 30% ferromagnetic particles using an (**a**) 8-magnet inductor and a (**b**) 16-magnet inductor.

Figure 13a,b shows the temperature evolution for the big and small needle-shaped composites elaborated with 25% and 30% iron oxide particles inductively powered by two types of inductors. The results were consistent to those obtained with the thermal camera and confirm the benefits of using a higher magnetic frequency to achieve better IH performance. Besides the frequency, the temperature variation of a ferromagnetic composite depends on other parameters such as particle content and volume of the sample. For better analysis of these relevant parameters, Table 1 summarizes the temperature change (∆T), where $∆T=T_{final}-T_{ambiant}$ based on the results of Figure 13. Interestingly, the 16-magnet inductor leads to a two-fold higher value of ∆T versus the 8-magnet one, which is coherent with the improvement in the 1.8-fold in frequency of the applied magnetic field. As expected, the 30% samples give a higher ∆T with respect to the 25% where the temperature ratio between these two cases reaches approximately 1.2. This value perfectly matches the proportion of the composite concentration (i.e., equal to 30 divided by 25). This result again demonstrates the linear relationship between the temperature variation as a function of the particle content (see also Figure 6b).

Finally, the volume of the big needle is 1.8 time higher than the small needle’s volume. This leads to a 1.8-fold increase in ∆T because the magnetic heat power (MHP) described in Figure 9b is considered to be dependent merely on the excitation frequency, the nature, and the content of the magnetic particles, but not on the geometry of composite. The experimental results showed a ratio of ∆T of around 1.4 to 1.6 times, which is lower than the expected theoretical value. This is likely due to a higher surface exchange with air of the bigger needle. This exchange leads to heat dissipation, resulting in slightly lower temperature.

COMSOL was used to study the thermal transfer of the needle-shaped composite exposed to an AC magnetic source, as described on [10]. In this model, the heat transfer coefficient of convection in air is chosen equal to 20 W m^–2^ K^–1^ [22]. As reported on [23], the specific heat capacity (*C_p_*) of a composite can be fitted by Equation (3), and is actually equal to the weighted average *C_p_, _composite_* of each constituent heat capacity in the case of an isotropic material with constant pressure and volume (negligible thermal expansion) with no local strain or stress [24].

(3) $$C_{p,composite}=(1-w)C_{p,ABS}+wC_{p,\;Fe_{3}O_{4}}$$

where *w* denotes the weight concentration of the iron oxide; $C_{p,ABS}$ and $C_{p,\;Fe_{3}O_{4}}$ are, respectively, the specific heat capacity of the ABS polymer (≈1800 J kg^−1^ K^−1^) and the Fe_3_O_4_ particles (≈450 J kg^−1^ K^−1^). Accordingly, the *C_p_* coefficient decreases from 1800 J kg^−1^ K^−1^ to 770 J kg^−1^ K^−1^ with magnetic fraction in the polymer at the volume concentration of 30%.

On the other hand, the thermal conductivity of composite (*λ*_composite_) tends to enhance when increasing the ferromagnetic content. The Maxwell model is tailored for composites composed of a dispersed and a continuous phase, and gives the following expression for the thermal conductivity in the case of dispersed iron oxide particles in an ABS polymer matrix [25]:

(4) $$\lambda_{composite}=\lambda_{ABS}\frac{\lambda_{Fe_{3}O_{4}}+2\lambda_{ABS}+2x(\lambda_{Fe_{3}O_{4}}-\lambda_{ABS})}{\lambda_{Fe_{3}O_{4}}+2\lambda_{ABS}-x(\lambda_{Fe_{3}O_{4}}-\lambda_{ABS})}$$

where *x* denotes the volume concentration of the iron oxide; $\lambda_{p,\;Fe_{3}O_{4}}$ and $\lambda_{ABS}$ are the thermal conductivity of the ABS polymer (≈0.3 W m^−1^ K^−1^) and the Fe_3_O_4_ particles (≈0.85 W m^−1^ K^−1^). The thermal conductivity of the sample filled with 30% vol of Fe_3_O_4_ was found equal to approximately 0.43 W m^−1^ K^−1^

**Figure 14:**
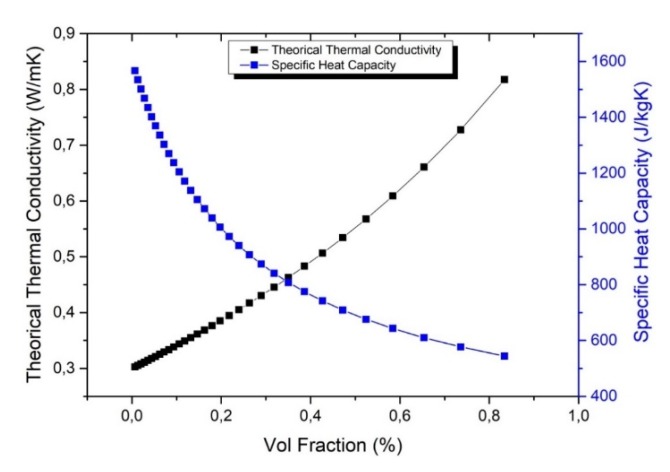
Thermal conductivity (black line) and specific heat (blue line) as a function of iron oxide content.

Figure 14 represents the evolution of the thermal conductivity and the specific heat capacity versus the volume concentration of the magnetic composite based on the theoretical model of Equations (3) and (4).

**Figure 15:**
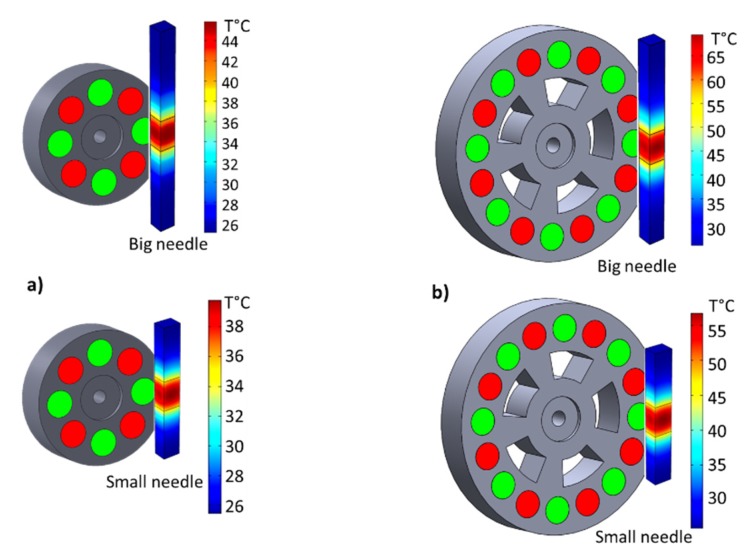
Spatial temperature evolution of two needle composites filled with 30% iron oxide using (**a**) an 8-magnet inductor and (**b**) a 16-magnet inductor.

**Figure 16:**
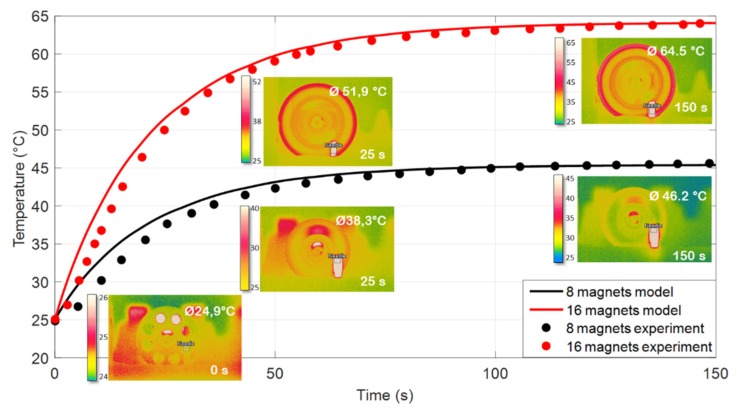
Theoretical and experimental temperatures as a function of time for the big needled composites filled with 30% vol. iron oxide excited by two different inductors.

**Figure 17:**
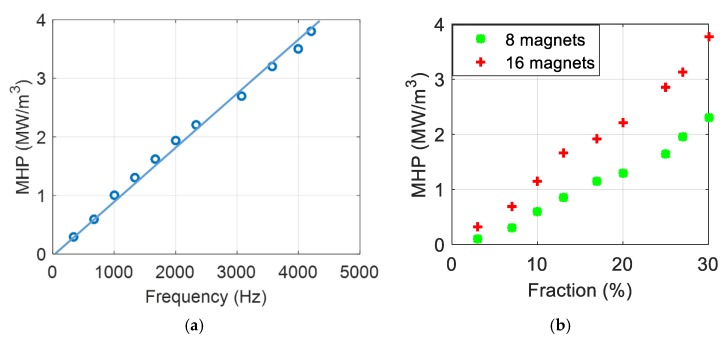
(**a**) Magnetic heat power (MHP) versus frequency of a composite doped with 30% vol. of iron oxide particles. (**b**) MHP versus fraction content of magnetic composite with two different frequency excitations.

Figure 15a,b shows the spatial evolution temperature of these two samples incorporated with 30% iron oxide excited by the 8- and 16-magnet inductors, respectively. Both needle-shaped composites have analog thermal transfer profiles where the temperature at the center close to the permanent magnet reached a maximum value and gradually decreased towards both sides further from the center. As expected, the 16-magnet inductor leads to a higher heating temperature than the 8-magnet system. Similar behavior has been obtained for the other samples doped with different Fe_3_O_4_ contents. Figure 16 described the simulation and experimental temperatures of the big needle composites driven under 2300 Hz and 4200 Hz AC magnetic power. The same trend was recorded in the case of the small needle, but its result was not shown here for a sake of simplicity. Excellent agreement between the theoretical and the empirical temperatures has been achieved, reflecting high reliability of the proposed thermal transfer model together with an estimation of the magnetic heat power (MHP) that is quasi-linear to the excitation frequency as well as the magnetic concentration of the fabricated material (Figure 17).

## 5. Conclusions

This paper reports a significant improvement of the low-frequency induction heating effect by increasing the frequency of the AC magnetic excitation as well as the iron oxide content dispersed inside the ABS thermoplastic. Experimental and simulation results showed the feasibility of inductively heating the ferromagnetic composite to 100 °C, which is close to the target temperature imposed by the venous insufficiency procedure. It has been highlighted that the heating efficiency not only depends on the frequency excitation and the magnetic concentration of samples but also on the nature of particles as well as the dimensions of the composite. To fit with medical tools, the ferromagnetic devices were elaborated to a needle-like shape, and great IH improvement was achieved based on the new inductor design comprising a doubling of permanent magnets compared to the former one. With the aim of boosting IH performance, future research will optimize the material processes, and magnetic excitation to fulfill specific requirements of thermal endovenous treatments, such as fast response time and precise/homogeneous heating. An alternative aspect of this work focusses on enhancing the mechanical properties of the magnetic composite to be adaptable to additive manufacturing and 3D printing. To confirm the reliability of the proposed approach for real clinical environments, further in vitro and in vivo tests will be considered in our future work.

## Figures and Tables

**Table 1:** Temperature change (∆T) of the big and small needles doped with 25% and 30% iron oxide based two different magnetic sources.

	8-Magnet Inductor		16-Magnet Inductor	
**Big Needle**	18 °C	21 °C	35 °C	40 °C
**Small Need**	11 °C	14 °C	25 °C	30 °C
